# Competency in medical history taking—the training physicians’ view

**DOI:** 10.1007/s00508-018-1431-z

**Published:** 2018-12-19

**Authors:** Tamara Seitz, Barbara Raschauer, Angelika S. Längle, Henriette Löffler-Stastka

**Affiliations:** 1 Social-Medical Center South, Vienna, Austria; 2 Dept. of Psychoanalysis and Psychotherapy, Medical University Vienna, Währingerstraße 18–20, 1090 Vienna, Austria; 3 Postgraduate Unit/Teaching Center, Medical University Vienna, Vienna, Austria

**Keywords:** Medical history, Anamnesis, Doctor-patient communication, Empathy, Medical students

## Abstract

**Background:**

Because effective communication skills are crucial for every physician, this study evaluated students’ competence to take a sufficient medical history. This ability was measured via reports from the students’ supervising and training physicians.

**Methods:**

A total of 24 physicians from several different departments were interviewed‚ and a stratification of medical specialties was performed due to the current supply-relevant number of medical doctors in the country. A qualitative content analysis was then performed.

**Results:**

The analysis revealed the students’ lack of expertise and ability to take a structured and complete medical history. Additionally, the lack of students’ engagement was criticized by the training physicians. An insufficiency of student supervision was also shown as only half of the departments have a fixed supervisor for the students during the internship.

**Conclusion:**

The data showed the need for a refinement of the training of communicative skills at university and the practical training at hospitals.

## Introduction and objectives

Effective communication skills are crucial for every physician. For example, from only one patient interview, a doctor with adequate communication skills could make a correct diagnosis with 76% accuracy [1]. Additionally, good communication skills and a high level of empathy predicts a higher level of patient satisfaction [2, 3], which positively influences patients’ adherence to therapy, keeping appointments‚ and adds to improving their health outcomes [2]; however, the majority of patients in the USA [4, 5] as well as in Europe [4] were shown to be dissatisfied with the quality of the doctor-patient communication. Specifically, the decreasing patient-centered communication was criticized. This may explain the increased frequency of errors made by physicians [6] and the increasing number of medical malpractice cases [7] and court cases [8]. Thus, the further development and improvement of communication skills training for medical students should be a primary goal for any medical university. Medical students have their first communicative training courses during university. Afterwards these skills are used during practical internships across different departments. In Austria, like in many other countries, only one physician per department is responsible for the students’ supervision and learning progress for the duration of the internship. This study investigated students’ competencies in taking a medical history as evaluated by their supervising physician. Furthermore, it was assessed which aspects of taking a medical history are important to experienced physicians.

## Methods

The study was performed with qualitative methods using a structured content analysis concept. This approach allows the participants to describe their experiences in their own words. Qualitative analysis requires informative cases instead of a sample size calculation including the statistical power. To generate representative informative cases, the method of target-oriented stratification [9] was applied. To ensure content target-orientation and sufficient variation range, preselected parameters from prior studies [10] were utilized and resulted in a minimum sample size of 10 interviews. To achieve a representative sample at least 20 supervising physicians from at least 5 different hospitals were to be interviewed. These supervising physicians were selected from a representative range of medical specializations. The focus was on covering the most central specializations, such as internal medicine, surgery, primary surgical subjects (e. g. gynecology), primary non-surgical subjects (e. g. neurology), psychiatry and pediatrics. All the supervisors had at least 10 years of clinical experience and were registered and certified as supervisors for students attending internships at the hospital.

### Measurements

A structured interview framework was created based on the critical incident technique [11]. The interview guidelines were developed as a teamwork project, consisting of experts from different medical specialties and domains (neurology, psychiatry, forensic medicine, general and family medicine, medical psychology) and psychologists (clinical and healthcare psychology, medical education). All participants possessed expertise in medical education.

### Procedure

Potential participants were first identified and then approached by telephone or email. They were briefed about the purpose of the study and if they agreed to participate an appointment was made. At the meeting the participant was first ensured of the anonymity of the answers and that the audio files would be removed after use. The interview was then performed and recorded after obtaining permission from the participant. The interviews were conducted in a private and quiet place. They lasted for 30 min on average. Interviews began with general topics, such as the physician’s specialization and clinical and teaching experience. Afterwards, questions about the students’ tasks and supervision during the internship were asked. The aims of taking a medical history (the aims of both the students and the supervising physicians) were evaluated with open questions such as: what should be the aims of taking medical history by students? What are the aims of taking medical history by yourself? Which aspects would please/displease you?

The supervisor’s assessment of student ability in performing an adequate anamnesis was initially addressed with a closed question: do you think that students doing their internship in your department are well prepared for performing an adequate anamnesis? Open questions were then asked to let the supervisors talk about the positive and negative qualities that students showed during their internship.

### Analysis

After transcription of the interviews, analysis of the data was performed by qualitative content analysis [12] and subsequent deductive [13] application of categories [14]. The text was at first examined for meaning units consisting of statements relevant to the aims of the study. These meaning units were shortened and summarized so that only the core message remained. The summarized meaning units were assigned codes and sorted into main categories and subgroups. The codes in the same main category were compared for similarities and differences regarding meaning and content to generate subcategories. Data was analyzed by two authors independently and after discussion of each result at a meeting a final version was agreed upon.

## Results

In total 136 interview requests were made, 17.6% (*n* = 24) agreed to the interview, 33 refused and 58% did not respond. More men were in the group of participating persons (16 vs. 8), whereas the gender distribution was in balance in the group of all primarily asked persons. The mean age (46 vs. 44 years) and the teaching experience was similar in the group of participating persons and the group of non-participating persons. All together 24 interviews in 24 different departments across 8 different hospitals located in Vienna and its surrounding suburbs were performed. Of the attending physicians six were from the field of surgery, three from internal medicine, four from psychiatry and child and adolescent psychiatry, five from gynecology and obstetrics, one from neurology, one from dermatology, one from otorhinolaryngology, one from anesthesiology, one from pediatrics and one from dentistry. Each of them had over 10 years of clinical experience, conducted more than 500 anamnestic encounters per year and was registered as a supervisor for students attending internships at the hospital.

### Contextual results

#### Students’ tasks during the internship

The interviewed physicians mentioned that students should take medical histories (*n* = 10) and perform practical clinical tasks (*n* = 9), such as medical examination (*n* = 6), taking blood (*n* = 2) and assisting in the operating room (*n* = 2). All physicians stated that they supervised students from the third year to the end of university studies. No accumulation of students of a special year was described.

#### Supervision of the students

Introductory conversations (defined as those between the student and supervisor that took place at the beginning of the internship) were held in only one department; however, in one third of the departments a concluding conversation to give the student feedback was also included. Only three departments reported wanting feedback from the student. Fewer than half of the departments have one or two assigned supervisors for each student for the whole period of the internship while the other departments have changing supervisors or none at all. Only four departments offer regular training and lectures for the students.

#### Evaluation

Of the interviewed physicians 54% (*n* = 13) stated that students are well prepared for taking a medical history; however, 23.8% (*n* = 5) noticed differences between the students’ performances, while 12.5% (*n* = 3) evaluated students’ ability to take a medical history as “bad”. No relationship was shown between age, gender or extent of clinical or teaching experience of the interviewed physician and evaluation.

### Qualitative analysis

A total of five main categories were found:

1. Medical content-related level
2. Setting and structure
3. Relationship level
4. Personal factors
5. Linguistic interaction

#### Supervisors’ aims of taking a medical history

If the supervising physicians are taking the medical history by themselves, the aspects of the relationship level are most important to them and two thirds discussed the importance of building a relationship of trust between the patient and the physician. Furthermore, exhibiting emotional skills such as empathy and appreciation (e. g. showing interest, being polite and patient) towards the patients was mentioned by many supervisors as a crucial component of the relationship. Of the supervisors‚ two stressed the importance of giving the patients enough information to reassure them and answer their questions. Half of the interviewed physicians stated that an aim of taking medical history is to collect enough information to determine the next diagnostic steps and to find possible diagnoses and therapies. Approximately one third mentioned aspects of time and structure: 21% strove for an anamnesis with enough time for the patient, 8% preferred to do a structural and time-efficient anamnesis. Interestingly, the physicians who were willing to invest more time in the anamnesis were from internal medicine or neurology/psychiatry while those who preferred the time-efficient anamnesis, were from surgery. Of the physicians‚ two also stated that it is crucial to have an undisturbed doctor-patient-conversation. Linguistic-interactive aspects were mentioned by one fifth, like having an open talk at the beginning and letting the patient talk without interruption, avoidance of medical terminology and adjustment to the patient’s level of knowledge and education. The detailed breakdown is shown in Table 1.

**Table 1:** Aims of taking a medical history (view of the supervisor)—Ideal type

	*n* (%)
*Relationship level*	14 (58)
Relationship of trust	8 (33)
Empathy	5 (21)
Appreciation	5 (21)
Information transfer	2 (8)
*Medical content-related level*	12 (50)
Diagnostic steps and treatment	7 (29)
Information collection	5 (21)
Diagnosis	2 (8)
*Setting and structure*	7 (29)
Enough time	5 (21)
Time-efficiency	2 (8)
Undisturbed room/setting	2 (8)
*Linguistic interaction*	5 (21)
Open discussion	2 (8)
Adjustment to the patient	2 (8)
Avoidance of medical language	1 (4)

#### Aims of taking a medical history by students from the supervisor’s point of view

Almost every one of the interviewed supervisors (96%) reported students attempting to take a medical history with aspects from the medical content-related level. The majority emphasized the importance of collecting information to deduce further diagnostic steps and therapy. About half of the supervisors mentioned the importance of current symptoms, others attached importance to the remaining medical history, such as chronic diseases, family history, 21% mentioned aspects of the relationship level, such as showing empathy and interest or being polite and friendly while only two discussed the importance of a structural and time-efficient anamnesis. The detailed breakdown is shown in Table 2.

**Table 2:** Aims of taking a medical history by students from the point of view of supervisors

	*n* (%)
*Medical content-related level*	23 (96)
Diagnostic steps and therapy	14 (58)
Current symptoms	11 (46)
Chronical diseases	10 (42)
Previous medical reports	7 (29)
Psychosocial history	6 (25)
Information collection	4 (17)
Medication history	3 (13)
Family history	2 (8)
*Relationship level*	5 (21)
Appreciation	4 (17)
Empathy	2 (8)
*Setting and structure*	2 (8)

#### Evaluation of the student by the supervisors

Several physicians criticized the students’ lack of experience (*n* = 7) and expertise (*n* = 7). While two supervisors positively mentioned the adequate and careful documentation, three criticized the lack of it. Of the questioned physicians one third said that students did not get all the important information in the process of taking a medical history while only one stated that they did. Physicians positively mentioned students’ empathy (*n* = 4) and appreciation (*n* = 4) toward the patient and students’ engagement (*n* = 1); however, more physicians talked about students’ lack of engagement (*n* = 5) and appreciation (*n* = 5). Approximately 17% of the physicians liked the structure of the students’ anamnesis but more than twice as many felt students had no structure at all (*n* = 8) and two physicians positively mentioned that students take enough time for the patient. Of the supervisors one described the students’ self-confidence in a positive way, while three described it in negative terms (i. e., too much or too little self-confidence). The detailed breakdown is shown in Table 3.

**Table 3:** Evaluation of students by the supervisors

Positively associated *n* (%)		Negatively associated *n* (%)
*Medical content-related level*		
2 (8%)	Documentation	3 (13%)
–	Experiences	7 (29%)
–	Expertise	7 (29%)
2 (8%)	Information collection	8 (33%)
*Personal factors*		
1 (4%)	Self-confidence	3 (13%)
*Relationship level*		
4 (17%)	Empathy	–
1 (4%)	Engagement	5 (21%)
4 (17%)	Appreciation	5 (21%)
*Setting and structure*		
4 (17%)	Structure	8 (33%)
2 (8%)	Time	–

## Summary and Resume

Interestingly, the interviewed physicians attached most importance to having a good relationship with the patient; however, they stated that the main aim for students should be to collect enough data about the patient’s current symptoms and medical history. Presumably, they think that the basis of taking an adequate medical history should be learned and mastered by students first, before focusing on more advanced skills, such as showing empathy towards the patient or building a relationship of trust.

Although about one half of the supervisors rated students’ skills of taking medical a history as sufficient, they pointed out a large range within the students. Also, 13% of the interviewed physicians stated that students were not prepared to make an adequate anamnesis at all and 30% claimed that students are not able to collect all important information, especially due to the lack of expertise and experience. The lack of expertise may be responsible for an unawareness of what to ask and for the inability to make a structured and complete anamnesis. Therefore, the implementation of medical field-specific communication courses at medical universities is suggested. It is assumed that the integration will lead to a transfer from students’ declarative to associative and procedural knowledge. For example, the training might start with the transmission of declarative knowledge with textbooks, frontal lectures and group seminars, followed by case-based e‑learning programs and simulated patient contact. Previous literature has demonstrated that training with standardized simulated patients (SPs) in medical education is a valuable tool [13], correlating with a high learner satisfaction [15] and an improvement in learners’ understanding of certain topics and skills [16]. The simulated patients might play different settings, so the student gets trained to ask the right questions during a medical emergency, while taking a preoperative anamnesis or in telling the patient bad news. Different medical fields, such as internal medicine, pediatrics or psychiatry should be integrated as well.

Regarding students’ behavior towards the patient, physicians’ opinions diverged widely. Some commended the students’ empathy towards the patient while others emphasized the lack of these qualities. A few noted that students showed too much or too little self-confidence while talking with a patient. A possible explanation could be that the different departments have students with different study progress and clinical experience. The literature shows that the majority of the students reported insecurity and a feeling of too much responsibility while working with patients for the first time and they often felt overloaded while handling their feelings [17]. Insecurity and arousal might have different consequences on performance, a phenomenon known as the Yerkes-Dodson law [18]. On the one hand insecurity might be a common human reaction while doing something new. Thus, the advantage of doing something for the first time could be that stress leads to increased attention helping to focus and, in this case, encouraging the student to do an interview properly and with high accuracy. On the other hand, if insecurity is too high (due to a lack of knowledge of how to do an interview, for example) the arousal might interfere with or even block the student’s ability to perform an interview. Further insecurity and overload might lead to social withdrawal and prevent future prosocial behavior [17, 19]. Therefore, it is suggested that students work out guidelines, in terms of a structured interview. This structure primarily provides support for future doctors, which leads to security and an appropriate amount of arousal. Ideally this increases attention and helps the student to focus on the task. Furthermore, guidelines for a structured interview would help students achieve the main aim of an anamnesis: collecting data about patient’s current symptoms and medical history. To increase student motivation the importance of such an interview should be emphasized. With more practice, more capabilities and knowledge about interviewing, trust in oneself will undoubtedly improve. This increases the possibility of a more open interview, allowing for more empathy and correspondingly, to the most important factor stressed by the interviewed physicians: the relationship level.

Several physicians criticized students’ lack of engagement during the internship. A possible explanation is a lack of supervision. The data showed that less than half of the departments, where the interviewed physicians were working, had at least one fixed supervisor (sometimes two) for each student. Additionally, there was a lack of regular feedback to students. In a recently published study, medical students were asked to evaluate the quality of internships in Vienna and surrounding areas [20]. The results corresponded with this study, showing that regular supervision and feedback are missing. Furthermore, students stated that they did not feel appreciated or integrated into the team. The literature showed that integration into the team and regular supervision/feedback are crucial for intrinsic motivation and sufficient learning [21]. To improve the supervision and integration into the team, the supervisors’ workloads and performances may be improved by financial incentives or allotted time off in compensation, or professional incentives concerning their teaching careers. Students should evaluate the supervisor after the internship. The supervisor might be replaced after receiving multiple negative reviews.

### Limitations

Even though the interviewed physicians stated that they supervise students from all years of university, it is to be expected that some departments have more advanced students than others. Another limitation is the heterogeneity of physicians and the retrospective design. The described lack of supervision might limit informative value of the interviewed physicians.

## Conclusion

This study showed that medical students are on average well prepared for taking a medical history; however, a lack of medical field-specific knowledge was shown, leading to an inability to make a structured and complete anamnesis. Furthermore, the lack of students’ engagement was also criticized. After graduation, the main challenge for physicians seems to be in responding to patients’ individual needs and to act empathically and appreciatively. How to improve medical education regarding the doctor–patient conversation should be examined in further studies.

## References

[1] Peterson MC, Holbrook JH, Von Hales D (1992). Contributions of the history, physical examination, and laboratory investigation in making medical diagnoses. West J Med.

[2] Schmid-Mast M, Kindlimann A, Hornung R (2004). How gender and communication style of physicians affect patient satisfaction: the little difference. Praxis.

[3] Derksen F, Bensing J, Lagro-Janssen A (2013). Effectiveness of empathy in general practice: a systematic review. Br. J. Gen. Pract..

[4] Langewitz W, Conen D, Nübling M (2002). Communication matters—deficits in hospital care from the patients’ perspective. Psychother Psychosom Med Psychol.

[5] Sator M, Gstettner A, Hladschik-Kermer B (2008). Doctor-patient-communication in an oncological outpatient department. A linguistic study of communication problems. Wien Klin Wochenschr.

[6] Chen RC, Clark JA, Manola J (2008). Treatment ‘mismatch’ in early prostate cancer: Do treatment choices take patient quality of life into account?. Cancer.

[7] Wienke A (2013). Briefing and accusation of medical malpractice—the second victim. Laryngorhinootologie.

[8] Tamblyn R, Abrahamowicz M, Dauphinee D (2007). Physician scores on a national clinical skills examination as predictors of complaints to medical regulatory authorities. JAMA.

[9] Sandelowski M (2000). Combining qualitative and quantitative sampling, data collection, and analysis techniques in mixed-method studies. Res Nurs Health.

[10] Seitz T, Turk BR, Löffler-Stastka H (2015). They’ve talked the talk, but can they walk the walk? Physician-educator evaluation of medical students’ communication skills in clinical practice—a pilot study. Adv Soc Behav Sci.

[11] Flanagan JC (1954). The critical incident technique. Psychol Bull.

[12] Hsieh HF, Shannon SE (2005). Three approaches to qualitative content analysis. Qual Health Res.

[13] Levine A, Swartz MH (2008). Standardized patients: the ‘other’ simulation. J Crit Care.

[14] Mayring P (2000). Qualitative content analysis.

[15] McNaughton N, Ravitz P, Wadell A (2008). Psychiatric education and simulation: a review of the literature. Can J Psychiatry.

[16] Xie H, Liu L, Wang J (2015). The effectiveness of using non-traditional teaching methods to prepare student health care professionals for the delivery of mental state examination: a systematic review. JBI Database System Rev Implement Rep.

[17] Pololi LP, Frankel RM, Clay M (2001). One year’s experience with a program to facilitate personal and professional development in medical students using reflection groups. Educ. Health. (Abingdon).

[18] Yerkes RM, Dodson JD (1908). The relation of strength of stimulus to rapidity of habit-formation. J Comp Neurol Psychol.

[19] Lamm C, Nusbaum HC, Meltzoff AN (2007). What are you feeling? Using functional magnetic resonance imaging to assess the modulation of sensory and affective responses during empathy for pain. PLoS ONE.

[20] Seitz T, Turk BR, Löffler-Stastka H (2016). Can we still stop the migration of physicians from Austria?—An evaluation of clinical internships done by students of the Medical University of Vienna. Wien Klin Wochenschr.

[21] Taylor DCM, Hamdy H (2013). Adult learning theories: implications for learning and teaching in medical education: AMEE guide no. 83. Med Teach.

